# On the propagation of waves in the atmosphere

**DOI:** 10.1098/rspa.2020.0424

**Published:** 2021-06

**Authors:** Adrian Constantin, Robin S. Johnson

**Affiliations:** ^1^ Faculty of Mathematics, University of Vienna, Oskar-Morgenstern-Platz 1, 1090 Vienna, Austria; ^2^ School of Mathematics, Statistics and Physics, Newcastle University, Newcastle NE1 7RU, UK

**Keywords:** atmospheric waves, asymptotic methods, differential equations

## Abstract

The leading-order equations governing the unsteady dynamics of large-scale atmospheric motions are derived, *via* a systematic asymptotic approach based on the thin-shell approximation applied to the ellipsoidal model of the Earth’s geoid. We present some solutions of this single set of equations that capture properties of specific atmospheric flows, using field data to choose models for the heat sources that drive the motion. In particular, we describe standing-waves solutions, waves propagating towards the Equator, equatorially trapped waves and we discuss the African Easterly Jet/Waves. This work aims to show the benefits of a systematic analysis based on the governing equations of fluid dynamics.

## 1. Introduction

Waves play a fundamental role in the development and evolution of our atmosphere; it is generally accepted, for example, that Rossby waves are the most important large-scale waves (controlling the weather in the mid- and higher latitudes), although gravity (buoyancy) waves and Kelvin waves are also significant. Of course, there are many other ingredients that contribute to the motion of the atmosphere, both locally and globally; here, we focus on the more familiar and well-documented wave-like motions. The complexities of the unsteady flows, including wave motions, suggest—apparently—that these cannot be systematically extracted from the full set of governing equations. Indeed, the familiar way forward is to simplify, to extreme levels, both the geometry and the assumed background state (which is then perturbed to produce wave motions, for example). Such an approach ignores the detailed structure of any ambient state of the atmosphere, which, in any event, must be regarded as representative of suitable averages over time and space, in order to produce fairly simple and accessible theoretical treatments.

It would seem that the conventional analyses have been developed because, it has been argued, neither the correct almost-spherical geometry nor a realistic background state could be incorporated within the system of relevant governing equations. Retaining the full nature of the Earth’s curved-space geometry is essential for large-scale atmospheric flows. To go beyond the limitations of the flat geometry of the *f*-plane approximation, the typical approach consists in invoking a weak contribution from the curvature by using the *β*-plane approximation (see [1]). However, in contrast to the *f*-plane approximation, the *β*-plane approximation fails to represent a consistent approximation to the governing equations for geophysical flows at mid-latitudes and in polar regions; see [2]. A further typical simplification in research investigations is to ignore the fine structure of the density variation by introducing weighted averages or by relying on the Boussinesq approximation.

As demonstrated by [3] in the case of steady flow, a detailed and extensive description of the motion in an atmosphere that envelopes an ellipsoidal model of the geoid, superimposed on a stationary but variable background state, can be formulated and solved. We now show that the same approach can be adopted when time dependence is added to the system, which, in this initial phase of the development of a coherent mathematical description, is based on an idealized model of the atmosphere. It is therefore appropriate to set aside some of the more realistic but complicating properties of the atmosphere as we observe it, such as turbulence, instabilities and convection processes (see [4]). The plan for the future is to build on this robust theoretical basis, progressively adding more of the important elements that play a role in our atmosphere. Necessarily, our approach differs quite markedly from the more familiar developments that have been employed to describe the appearance and properties of waves in the atmosphere. At the heart of our method is the careful construction of an asymptotic solution driven primarily by the thin-shell approximation, retaining all the other physical attributes of the problem (such as a model for the accurate representation of the oblate-spherical geometry, variable background state, variable density and viscosity and general heat sources). Note that, because the oblateness is only slight for the Earth, it is convenient to incorporate this property as a small correction to the otherwise spherical coordinate system; importantly, we show that this contribution to the geometry uncouples from the thermodynamics and dynamics of the atmosphere, at leading order. The upshot is that the unsteady motion sits on a realistic background state, which results in a very different structure when, for example, waves are included. In particular, we see that any unsteady motion—especially waves—appear at the same order as the underlying dynamic-thermodynamic balance: they do not constitute small perturbations of some uniform (constant) state. This is an important and fundamental difference, which, when coupled with the overarching assumption of a thin-shell geometry—the only simplifying assumption we make for the geometry—for the atmosphere over the surface of the Earth, produces a novel approach to these problems. The result, we claim, provides a far more mathematically consistent presentation of the underlying structure of the atmosphere than anything attempted hitherto, but necessarily it provides a different-looking description for wave propagation even if, superficially, it is very familiar. Furthermore, the resulting system of equations is derived from, and is consistent with, a set of governing equations for the general fluid dynamics of the atmosphere.

The plan is to outline the derivation of the governing equations—the details can be found in [3]—and then we carefully describe how the unsteady motion, with waves, can be accommodated. The resulting system will be analysed and various special cases investigated. Note that throughout the last decades considerable insight into atmospheric flows has been gained by studying the plethora of empirical models that typically rely on observational data and heuristic simplifications of the governing equations. As for systematic asymptotic expansions (e.g. [5,6] and publications discussed therein), these do not start from the general set of fundamental, governing equations. However, all models that are consistent with physical reality must, ultimately, be reflections of a common framework because, underlying them all, is just one set of governing equations. This fact is a strong motivation to derive a leading-order generic system of (reduced) equations, based on reliable and transparent approximation procedures, as pursued in the present paper. Unsurprisingly, we recover qualitative features that are similar to those encountered in classical models, but there are a few important corrections to be noted, especially with regards to the dispersion relation of zonally harmonic waves and concerning the meridional decay rate of equatorially trapped waves (see §5). This situation is somewhat similar to that encountered in the investigation of Rossby waves: the beta-plane approximation was the step forward from the *f*-plane approximation that revealed to Rossby [7] the presence of these waves, but Haurwitz [8] extended the accuracy (especially with regard to the meridional extent of the wave) by taking the spherical shape of the Earth into account (see also [9,10]).

## 2. Governing equations

Following [3], we model the atmosphere as a compressible, viscous fluid (using the familiar Navier–Stokes and mass conservation equations of fluid dynamics, allowing for variable density and describing a viscous fluid), coupled to an equation of state and a suitable version of the first law of thermodynamics. The air is treated as a single-component gas, but with appropriate heating (both external, e.g. solar radiation, and internal, e.g. latent heat). This choice of model is the simplest that we can envisage at this initial phase of the investigation. There is no doubt that further refinements are possible, which, for example, could be used to provide a more accurate description of the background state of the atmosphere, e.g. by including the effects of moisture or any horizontal variation in the eddy viscosity. Such extensions are something for the future, but they will need to be incorporated within the framework of a general set of governing equations that describe the fluid that represents the atmosphere.

**Figure 1.**
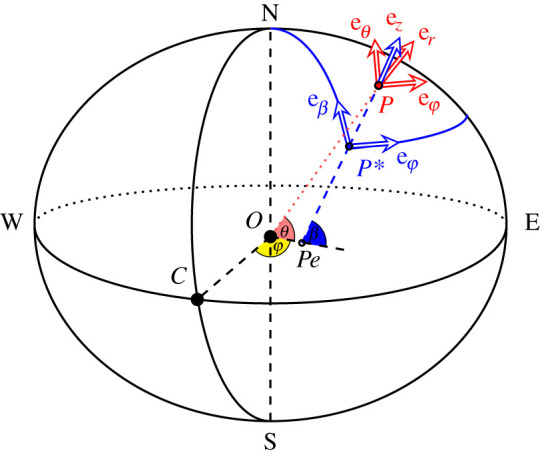
Away from the polar axis, we represent a point *P* in the atmosphere using the hybrid spherical-geopotential rotating coordinate system (*φ*, *θ*, *z*′), obtained from the spherical system $\left({\text{e}}_{\phi },{\text{e}}_{\theta },{\text{e}}_{r}\right)$ and the geopotential system $\left({\text{e}}_{\phi },{\text{e}}_{\beta },{\text{e}}_{z}\right)$. Here, *φ* and *θ* are the longitude and geocentric latitude of *P*, respectively, *β* is the geodetic latitude of the projection P* of *P* on the ellipsoidal geoid and **e**_*z*_ points upwards along the normal P*P to the geoid (which intersects the equatorial plane in the point *P*_*e*_). The unit vectors $\left({\text{e}}_{\theta },{\text{e}}_{r}\right)$ are obtained by rotating the unit vectors $\left({\text{e}}_{\beta },{\text{e}}_{z}\right)$ by the angle (*β* − *θ*), in the plane of fixed longitude *φ*. (Online version in colour.)

The shape of the Earth’s sea-level geopotential surface—essentially an oblate spheroid—is well-known from satellite data. It is usual, in the atmospheric sciences, to approximate this oblate spheroid by an ellipsoid obtained by rotating an ellipse, whose centre coincides with the centre of the Earth, about its semi-minor (polar) axis (of length *d*_*P*_′ ≈ 6357 km), with a semi-major (equatorial) axis of length *d*_*E*_′ ≈ 6378 km. (We use primes to denote physical [dimensional] variables; these will be removed when we introduce a suitable non-dimensionalization.) In particular, this accurately accounts for the Earth’s equatorial bulge of approximately 21 km and, furthermore, this ensures that the largest departure from the true shape is about 100 m (as a depression to the south of India); see [11]. The Cartesian coordinates (*X*′, *Y*′, *Z*′) of the ellipsoid, with the origin at the Earth’s centre and the vertical axis through the North Pole, can be expressed in terms of the longitude and geodetic latitude *φ* and *β*, respectively, by

$$(X^{\prime },Y^{\prime },Z^{\prime })=\frac{d_{E}^{\prime }}{\sqrt{1-e^{2}\sin^{2}\beta }}\;(\cos\beta \cos\phi ,\cos\beta \sin\phi ,(1-e^{2})\;\sin\beta ),$$

where

$$e=\sqrt{1-{\left(\frac{d_{P}^{\prime }}{d_{E}^{\prime }}\right)}^{2}}\approx 0.081$$

is the eccentricity. The coordinate system (*φ*, *β*, *z*′), *z*′ being the vertical distance up from the surface of the ellipsoid, is associated with the ellipsoid, which is rotating about its polar axis with (constant) angular speed $\Omega^{\prime }\approx 7.29\times 10^{-5}\;\text{rad}\;{\text{s}}^{-1}$. In this system, the unit tangent vectors at the surface of the ellipsoid are $\left({\text{e}}_{\phi },{\text{e}}_{\beta },{\text{e}}_{z}\right)$; **e**_*φ*_ points from West to East along the geodetic parallel, ${\text{e}}_{\beta }$ from South to North along the geodetic meridian and **e**_*z*_ points upwards (figure 1). The system is valid throughout the space, except along the direction of the polar axis, where this description fails because **e**_*φ*_ and ${\text{e}}_{\beta }$ are not well-defined at the two Poles. Note that the direction of apparent gravity (seen by an observer in the rotating frame) is normal to the surface of the ellipsoid, which is the isosurface of the geopotential that defines zero elevation; the geopotential being the sum of the Newtonian (gravitational) potential and the centrifugal potential due to the Earth’s diurnal rotation. The details of this geometric model are fully described in [3]. In summary, we transform from spherical coordinates, (*φ*, *θ*, *r*′), to the hybrid spherical-geopotential coordinates (*φ*, *θ*, *z*′), and couple this with a transformation of the velocity vector and the gravity term. Thus the components of these vectors, which are defined normal and tangential to the surface of the ellipsoid, are decomposed into components in the spherical system. As pointed out in [3], the advantage of using this spherical-geopotential hybrid rotating coordinate system rather than the conventional spherical potential approximation (described in [12]) is that the formulation retains the details of the curved-space geometry of the Earth and the leading-order (geometrical) correction terms apply to the background state of the atmosphere but do not interact (in the leading-order perturbation) with the dynamics of the atmosphere.

The relation between *r*′ and *z*′ is given by the identity

$$\frac{r^{\prime }}{d_{E}^{\prime }}=1+\epsilon z-\frac{1}{2}\;e^{2}\sin^{2}\theta -e^{4}\left(\frac{1}{2}-\frac{3}{8}\;\sin^{2}\theta \right)\sin^{2}\theta +\text{O}(e^{6},\epsilon e^{4},\epsilon^{2}e^{2},\epsilon^{3}),$$

with *z*′ = ε*z* (see below). Furthermore, we must use a non-dimensionalization that enables us to put the equations in a form that is relevant for a discussion of atmospheric flows. To do this, we introduce a scale length, which we take to be the maximum height of the troposphere, *H*′ (which is about 16 km at the Equator), and an associated speed scale $\Omega^{\prime }H^{\prime }$ (approx. 1.2 m s^−1^). Thus we define

$$\begin{array}{rl} & z^{\prime }=\epsilon z\;\text{where}\;\epsilon =\frac{H^{\prime }}{d_{E}^{\prime }},\;t^{\prime }=\frac{1}{\Omega^{\prime }}\;t,\;\rho^{\prime }={\bar{\rho }}^{\prime }\rho ,\;\mu^{\prime }={\bar{\mu }}^{\prime }\mu ,\\ & (u^{\prime },v^{\prime },w^{\prime })=\Omega^{\prime }H^{\prime }\;(u,v\cos(\beta -\theta )+kw\sin(\beta -\theta ),kw\cos(\beta -\theta )-v\sin(\beta -\theta )), \end{array}$$

where the over-bar denotes average (${\bar{\rho }}^{\prime }\approx 0.8\;\text{kg}\;{\text{m}}^{-3}$, ${\bar{\mu }}^{\prime }\approx 2\times 10^{-2}\;\text{kg}\;{\text{m}}^{-1}\;{\text{s}}^{-1}$ being density and dynamic eddy viscosity, respectively) and the time scale $1/\Omega^{\prime }$ measures time in units of about $3\frac{1}{2}\;\text{h}$ (this being appropriate for wave-like or other unsteady motions of the atmosphere). The velocity in spherical coordinates is (*u*′, *v*′, *w*′) and (*u*, *v*, *w*) is a non-dimensional velocity with *w* normal, and (*u*, *v*) tangential, to the ellipsoidal geoid, where

$$\beta -\theta =e^{2}\sin\theta \cos\theta +e^{4}\sin\theta \cos^{3}\theta -\epsilon e^{2}\;z\;\sin\theta \cos\theta +\text{O}(e^{6},\epsilon e^{4},\epsilon^{2}e^{2},\epsilon^{3}).$$

The constant *k* measures the size of the velocity component normal to the ellipsoid, and its choice controls the type of problem to be examined. In the case of thin-shell theory (i.e. small ε, as we have here), the choice consistent with the equations (and kinematic boundary conditions, for example) is *k* = ε; we use this identification hereafter.

The pressure (*p*′) and the temperature (*T*′) are correspondingly non-dimensionalized:

$$p^{\prime }={\bar{\rho }}^{\prime }{\left(\Omega^{\prime }d_{E}^{\prime }\right)}^{2}p,\;T^{\prime }=\frac{{\left(\Omega^{\prime }d_{E}^{\prime }\right)}^{2}}{R^{\prime }}\;T,$$

where R′ ≈ 287 m^2^ s^−2^ K^−1^ is the universal gas constant and ${\left(\Omega^{\prime }d_{E}^{\prime }\right)}^{2}/R^{\prime }\approx 800^{\circ }\;\text{K}$. The dynamic eddy viscosity is assumed to vary only in the radial direction (this being the choice that was made in [3]); the transformation from *r*′ to *z*′ then shows that, for the viscous terms that appear in the Navier–Stokes equation, we have

$$\mu =m(z)+\text{O}(e^{6},\epsilon e^{4},\epsilon^{2}e^{2},\epsilon^{3}).$$

We are now in a position to quote the governing equations, suitably approximated for small ε and small *δ* = R*e*^2^, this latter parameter measuring the effects of small deviations of the ellipsoid from the spherical. The equations follow directly from those derived in [3]; here, though, we retain the time dependence; the errors in each equation are recorded and we write out each component of the Navier–Stokes equation. We obtain

2.1 $$\begin{array}{rl} & \epsilon \rho \;\frac{\partial u}{\partial t}-2\epsilon \rho v\sin\theta =-\frac{1-\epsilon z+\delta \Delta }{\cos\theta }\;\frac{\partial p}{\partial \phi }+\frac{\epsilon }{R_{e}}\;\frac{\partial }{\partial z}(m\;\frac{\partial u}{\partial z})+\text{O}(\epsilon^{2},\epsilon \delta ,\delta^{2}), \end{array}$$

2.2 $$\begin{array}{rl} & \epsilon^{2}\rho \;\frac{\partial v}{\partial t}+2\epsilon^{2}\rho u\sin\theta +\epsilon \rho (1+\epsilon z-\delta \Delta )\sin\theta \;\cos\theta \\ & \;=-(1-\epsilon z+\delta \Delta )\;[\epsilon \;\frac{\partial p}{\partial \theta }+(2\delta \Delta +\delta^{2}D)\;\frac{\partial p}{\partial z}]\\ & \;-2\delta \Delta \rho g[1+\delta (\sin\theta +\cos\theta )\cos\theta -3\epsilon z]+\frac{\epsilon^{2}}{R_{e}}\;\frac{\partial }{\partial z}(m\;\frac{\partial v}{\partial z})+\text{O}(\epsilon^{3},\epsilon^{2}\delta ,\epsilon \delta^{2}), \end{array}$$

2.3 $$\begin{array}{rl} & -\epsilon \rho \cos^{2}\theta =-\frac{\partial p}{\partial z}-\rho g(1-2\epsilon z+2\delta \Delta )+\text{O}(\epsilon^{2},\epsilon \delta ,\delta^{2}), \end{array}$$

2.4 $$\begin{array}{rl} & \frac{D\rho }{Dt}+\epsilon \rho \left\{\frac{1}{\cos\theta }\left(\frac{\partial u}{\partial \phi }+\frac{\partial }{\partial \theta }\;(v\cos\theta )\right)+\frac{\partial w}{\partial z}\right\}=\text{O}(\epsilon^{2},\epsilon \delta ,\delta^{2}), \end{array}$$

2.5 $$\begin{array}{rl} & p=\rho T \end{array}$$

2.6 $$\begin{array}{rl} \;\text{and}\; & c_{p}\;\frac{DT}{Dt}-\epsilon \kappa \;\frac{\partial^{2}T}{\partial z^{2}}-\frac{1}{\rho }\;\frac{\text{D}p}{\text{D}t}=\epsilon \;Q(\phi ,\theta ,z;\;\epsilon ,\delta )+\text{O}(\epsilon^{2},\epsilon \delta ,\delta^{2}), \end{array}$$

where

$$\begin{array}{rl} & \frac{\text{D}}{\text{D}t}\equiv \frac{\partial }{\partial t}+\epsilon \;\frac{u}{\cos\theta }\;\frac{\partial }{\partial \phi }+\epsilon v\;\frac{\partial }{\partial \theta }+\epsilon w\;\frac{\partial }{\partial z},\\ & \Delta (\theta )=\frac{1}{2}\;\sin\theta \cos\theta ,\;D(\theta )=\left(1-\frac{3}{2}\;\sin^{2}\theta \right)\sin\theta \cos\theta . \end{array}$$

The additional parameters that we have introduced here, based on the (constant) average acceleration of gravity at the surface of the Earth (*g*′ ≈ 9.8 m s^−2^), the specific heat of air (*c*_*p*_′ ≈ 1500 m^2^ s^−2^ K^−1^) and the thermal diffusivity of predominantly dry air (*ν*′ ≈ 2 × 10^−5^ m^2^ s^−1^), are

$$\begin{array}{r} \begin{array}{ll} R_{e}=\frac{{\bar{\rho }}^{\prime }\Omega^{\prime }H^{\prime 2}}{{\bar{\mu }}^{\prime }}\approx 7\times 10^{5}, & g=\frac{g^{\prime }H^{\prime }}{{\left(\Omega^{\prime }d_{E}^{\prime }\right)}^{2}}\approx 0.72,\\ c_{p}=\frac{c_{p}^{\prime }}{R^{\prime }}\approx 5.25, & \kappa =\frac{\nu^{\prime }c_{p}^{\prime }}{R^{\prime }\Omega^{\prime }H^{\prime 2}}\approx 6\times 10^{-9}, \end{array} \end{array}$$

all O(1), i.e. held fixed as the limiting process is performed. Note that the appearance of large Reynolds number, *R*_*e*_, and small *κ*, suggests that additional approximations could be performed, but these are unnecessary (and would then require the introduction of viscous and thermal boundary layers, for example). We note that equation (2.6) is the first law of thermodynamics, with *Q* representing the (non-dimensional) heat sources (or sinks), expressing the change of total energy due to any heat exchanges. The second law of thermodynamics, setting limits for the transformations between heat energy and the sum of kinetic and potential energies (see [13,14]), will be not be of direct concern here: in the general discussion we do not, nor do we need to, specify the sources of the heat input since we do not address the issue of the climate being in a non-equilibrium thermodynamical state (see [15–17] for the challenges encountered in trying to exploit the second law to determine a lower bound to the entropy production). Furthermore, we choose to apply our results to regions of the Earth where the topography is relatively unchanging; the inclusion of significant orography, which will affect the bottom boundary condition and the local generation and structure of waves, is a refinement relegated to a future investigation.

## 3. Asymptotic structure of the solution

We seek a solution of (2.1)–(2.6) as an asymptotic expansion which begins

$$q(\phi ,\theta ,z,t;\epsilon ,\delta )∼q_{0}(\phi ,\theta ,z)+\epsilon \;q_{1}(\phi ,\theta ,z,t)+\delta \;{\hat{q}}_{1}(\phi ,\theta ,z,t),$$

where *q* (and correspondingly *q*_*n*_ and ${\hat{q}}_{n}$) represent each of the variables *u*, *v*, *w*, *p*, *ρ*, *T*. Furthermore, we assume that the boundary and initial conditions are consistent with these expansions, and that the heat-source term also follows this pattern. (The lack of any evident singularities in the velocity, temperature or pressure fields, providing that the neighbourhood of the Poles is avoided, and in the small-eccentricity corrections, indicates that this asymptotic structure is otherwise uniformly valid, i.e. higher-order terms remain small; more details are given in [3].) The leading-order problem (which is time-independent, being driven by ∂*ρ*_0_/∂*t* = 0 from (2.4)), then gives

$$\begin{array}{rl} & \frac{\partial p_{0}}{\partial \phi }=0;\;\rho_{0}\sin\theta \cos\theta =-\frac{\partial p_{0}}{\partial \theta };\;0=-\frac{\partial p_{0}}{\partial z}-\rho_{0}g;\;p_{0}=\rho_{0}T_{0};\\ & c_{p}L_{0}(T_{0})-\kappa \;\frac{\partial^{2}T_{0}}{\partial z^{2}}-\frac{1}{\rho_{0}}\;L_{0}(p_{0})=Q_{0}(\phi ,\theta ,z), \end{array}$$

where

$$L_{0}\equiv \left(\frac{u_{0}}{\cos\theta }\;\frac{\partial }{\partial \phi }+v_{0}\;\frac{\partial }{\partial \theta }+w_{0}\;\frac{\partial }{\partial z}\right);$$

note that the third equation above appears twice in the set (2.1)–(2.6). There is a solution of this system (see [3]) in which the temperature decreases linearly with height, independently of the velocity field (which nevertheless does appear in the equations at this order): the classical adiabatic model of the lower atmosphere. This corresponds to an external heat source of zero (*Q*_0_ ≡ 0): the heating is supplied by heat transfer from the surface of the Earth upwards into the atmosphere. (It should be noted, in our approach, that we determine the heat sources that are consistent with the motions that we describe; clearly such heat sources then drive the well-behaved flow properties. There is therefore no need to add general, auxiliary conditions on the background heating that would ensure the existence of solutions.) The resulting solution is

$$\begin{array}{rl} & T_{0}=A-\frac{1}{c_{p}}\;\left(gz-\frac{1}{2}\;\cos^{2}\theta \right);\;p_{0}=B\;{\left[A-\frac{1}{c_{p}}\left(gz-\frac{1}{2}\;\cos^{2}\theta \right)\right]}^{c_{p}};\\ & \rho_{0}=B\;{\left[A-\frac{1}{c_{p}}\left(gz-\frac{1}{2}\;\cos^{2}\theta \right)\right]}^{c_{p}-1}. \end{array}$$

We impose the boundary condition

$$T_{0}=T_{E0}\;\text{at}\;[z=0,\theta =\theta_{0}],$$

so that

$$A=T_{E0}-\frac{1}{2c_{p}}\;\cos^{2}\theta_{0};$$

the constant *B* is fixed by knowing the pressure (or density) at ground level at some *θ*. We have chosen to work with a background state which is steady; other choices are possible, e.g. via the velocity field (*u*_0_, *v*_0_, *w*_0_) or by allowing variations on a suitable, long time scale. As formulated here, we may choose to use the boundary conditions appropriate to the region of the Earth centred on (*z* = 0, *θ* = *θ*_0_) and to a particular season. This solution describes the background state, at leading order in ε and *δ*, for the troposphere, and it is only this section of the atmosphere that we shall discuss here; a related description for higher levels in the atmosphere can be found in [3].

The dominant effects of the ellipsoidal-geometry correction, as these distort the otherwise spherical solution, arise at O(*δ*); the equations at this order are

$$\begin{array}{rl} & 0=-\left(\frac{\partial {\hat{p}}_{1}}{\partial \phi }-\Delta \;\frac{\partial p_{0}}{\partial \phi }\right);\;({\hat{\rho }}_{1}+\Delta \rho_{0})\sin\theta \cos\theta =-\left(\frac{\partial {\hat{p}}_{1}}{\partial \theta }-\Delta \;\frac{\partial p_{0}}{\partial \theta }\right);\\ & 0=-\left(\frac{\partial {\hat{p}}_{1}}{\partial z}+\Delta \;\frac{\partial p_{0}}{\partial z}\right)-({\hat{\rho }}_{1}-2\Delta \rho_{0})g;\;\frac{\partial {\hat{\rho }}_{1}}{\partial t}=0;\;{\hat{p}}_{1}={\hat{\rho }}_{1}T_{0}+\rho_{0}{\hat{T}}_{1};\\ & c_{p}L_{0}({\hat{T}}_{1})-\kappa \;\frac{\partial^{2}{\hat{T}}_{1}}{\partial z^{2}}-\frac{1}{\rho_{0}}\;L_{0}({\hat{p}}_{1})+\frac{{\hat{\rho }}_{1}}{\rho_{0}^{2}}\left(v_{0}\;\frac{\partial p_{0}}{\partial \theta }+w_{0}\;\frac{\partial p_{0}}{\partial z}\right)={\hat{q}}_{1}(\phi ,\theta ,z,t); \end{array}$$

these equations have been simplified by using the equations that describe the leading order. The solution of this system simply produces a (uniformly valid) small adjustment to the background state, as described in [3]. However, the solution for these temperature, pressure and density perturbations is time-independent, and so the first law of thermodynamics (at this order) shows that, in order to maintain this structure, a heat source that moves with the fluid is required, e.g. some latent-heat input. We do not pursue the details of this contribution here because it uncouples from the leading-order time-dependent element of the motion (e.g. waves) that we want to investigate.

The important time-dependence appears at O(ε), which is also where the dominant contribution to both the dynamics and thermodynamics is evident; this general structure, for steady flow, is discussed in [3]. The resulting equations are

3.1 $$\begin{array}{rl} & \rho_{0}\;\frac{\partial u_{0}}{\partial t}-2\rho_{0}v_{0}\sin\theta =-\frac{1}{\cos\theta }\;\left(\frac{\partial p_{1}}{\partial \phi }-z\;\frac{\partial p_{0}}{\partial \phi }\right)+\frac{1}{R_{e}}\;\frac{\partial }{\partial z}\left(m\;\frac{\partial u_{0}}{\partial z}\right), \end{array}$$

3.2 $$\begin{array}{rl} & \rho_{0}\;\frac{\partial v_{0}}{\partial t}+2\rho_{0}u_{0}\sin\theta +(\rho_{1}+z\rho_{0})\sin\theta \;\cos\theta =-\left(\frac{\partial p_{1}}{\partial \theta }-z\;\frac{\partial p_{0}}{\partial \theta }\right)+\frac{1}{R_{e}}\;\frac{\partial }{\partial z}\left(m\;\frac{\partial v_{0}}{\partial z}\right), \end{array}$$

3.3 $$\begin{array}{rl} & -\rho_{0}\cos^{2}\theta =-\frac{\partial p_{1}}{\partial z}-g(\rho_{1}-2z\rho_{0}), \end{array}$$

3.4 $$\begin{array}{rl} & \cos\theta \;\frac{\partial \rho_{1}}{\partial t}+\frac{\partial }{\partial \phi }\;(\rho_{0}u_{0})+\frac{\partial }{\partial \theta }\;(\rho_{0}v_{0}\cos\theta )+\frac{\partial }{\partial z}\;(\rho_{0}w_{0}\cos\theta )=0, \end{array}$$

3.5 $$\begin{array}{rl} & p_{1}=\rho_{0}T_{1}+\rho_{1}T_{0}, \end{array}$$

3.6 $$\begin{array}{rl} \text{and}\;\; & c_{p}\;\frac{\partial T_{1}}{\partial t}-\frac{1}{\rho_{0}}\;\frac{\partial p_{1}}{\partial t}=Q_{0}(\phi ,\theta ,z,t), \end{array}$$

where

$$Q∼Q_{0}(\phi ,\theta ,z)+Q_{0}(\phi ,\theta ,z,t)+\delta \;{\hat{q}}_{1}(\phi ,\theta ,z,t)$$

with *Q*_0_ ≡ 0 for the troposphere. For time-independent flows, equations (3.1)–(3.6) recover precisely those obtained in [3], but with (3.6) replaced by a more complete version of the first law of thermodynamics, which provides a mechanism for describing the heat sources associated with the motion. This difference arises because the leading terms in the time derivative vanish, so we must, perforce, go to the next order for the description of a steady dynamic-thermodynamic balance. The considerations in [3] describe the combined dynamic and thermodynamic properties of the steady troposphere; this further analysis has now demonstrated that, on time scales measured by $1/\Omega^{\prime }$, time dependence appears at this same order. We can expect, therefore, to find unsteady solutions which may not be wave-like, but also (superimposed on any such motions) wave solutions, both being the same size (in the ε sense) and both appearing in the dominant dynamic-thermodynamic structure of the atmosphere and, significantly, not as a small perturbation to a constant state.

Our main aim—to put the theoretical study of the motions of our idealized model of the atmosphere on a solid foundation based on the general equations of fluid mechanics—is motivated, in part, by the reported observations and data from field studies (e.g. satellite observations, regional and global weather data, together with weather forecasts, climate modelling and reanalyses). Indeed, atmospheric waves—we are interested here in stable waves—are typically identified experimentally from satellite observations, after having subtracted the background flow field *via* appropriate filtering methods. Most studies use spectral analysis to isolate various wave modes, seeking agreement with structures revealed in theoretical studies; see the discussion in [18]. The system (3.1)–(3.6) shows that a careful asymptotic derivation can accommodate a dynamic-thermodynamic balance, which encompasses more behaviour (and is readily accessible) than simply perturbing a constant zonal flow (as described in [1,19,20]). The latter oversimplification of the dynamics was driven, presumably, by the need to use a very restrictive form of heat forcing, coupled to the limitations of a simplistic model of the background state.

## 4. Development of the unsteady problem at leading order

The main aim in this initial investigation is to examine the unsteady problem associated with the dominant dynamic-thermodynamic balance, which arises at O(ε) in our idealized model of the atmosphere. (This model excludes, therefore, the role of turbulence, instabilities, etc.; it is planned to extend our approach by adding more extensive attributes of the atmosphere in subsequent studies.) In particular, we seek solutions—hopefully in a manageable form—of two types: unsteady, but not wave-like, and wave solutions. However, before we proceed, it is expedient to make some small adjustments to the presentation of equations (3.1)–(3.6); this will ease the later analysis.

First, writing

$$Q_{0}=\frac{\partial q_{0}}{\partial t}\;\text{with}\;q_{0}(\phi ,\theta ,z,t)=\int_{0}^{t}Q_{0}(\phi ,\theta ,z,s)\;\text{d}s,$$

equation (3.6) can be integrated to give

4.1 $$c_{p}T_{1}-\frac{1}{\rho_{0}}\;p_{1}=q_{0}(\phi ,\theta ,z,t)+A_{1}(\phi ,\theta ,z),$$

where *A*_1_(*φ*, *θ*, *z*) is an arbitrary function, fixed by the initial data on the perturbation temperature and pressure. Now from equation (3.3), we see that

4.2 $$p_{1}=\rho_{0}\{(gz^{2}+z\cos^{2}\theta )+g\Pi_{1}+B_{1}\},$$

where

4.3 $$\Pi_{1}=\int_{0}^{z}\left(\frac{q_{0}(\phi ,\theta ,\xi ,t)+A_{1}(\phi ,\theta ,\xi )}{T_{0}(\theta ,\xi )}\right)\;\text{d}\xi ,$$

and *B*_1_(*φ*, *θ*, *t*) is an arbitrary function determined, for example, by the perturbation pressure on the ground. The expression for $\Pi_{1}$ measures the total heat input, weighted with respect to the background temperature, from the bottom of the atmosphere upwards, and over time. The complete description of the thermodynamic properties of the atmosphere, at this order, is then obtained from (3.5) and (3.6) in the form

4.4 $$T_{1}=\frac{1}{c_{p}}\;\left(\frac{p_{1}}{\rho_{0}}+q_{0}+A_{1}\right),\;\rho_{1}=\frac{1}{c_{p}T_{0}}[(c_{p}-1)p_{1}-(q_{0}+A_{1})\rho_{0}],$$

where *p*_1_ is given by (4.2).

It is now convenient to introduce

4.5 $$F_{1}(\phi ,\theta ,z,t)=g\Pi_{1}(\phi ,\theta ,z,t)+B_{1}(\phi ,\theta ,t),$$

and first we show how the heat forcing (4.5) can be related directly to a familiar descriptor used in atmospheric studies. To do this, we introduce the potential temperature, $T=Tp^{-1/c_{p}}$, and the square of the (non-dimensional) Brunt–Väisälä frequency, *N*, where

4.6 $$N^{2}=\frac{g}{\epsilon }\;\frac{\partial \ln(T)}{\partial z};$$

see [19]. (We have introduced $\Omega^{\prime }\sqrt{d_{E}^{\prime }/H^{\prime }}$ for the non-dimensionalization leading to *N*.) This is used as a measure of the static stability of the atmosphere, the adiabatic perturbation of a fluid parcel about its equilibrium position being governed by

4.7 $$\frac{{\text{D}}^{2}}{\text{D}t^{2}}\;(\zeta )=-N^{2}(\zeta ),$$

for small vertical displacements *ζ*. Considering only the unsteady perturbation, i.e. the O(ε) terms (because the O(*δ*) terms uncouple from any wave-like motion and contribute only to the background state), we have

$$T=Tp^{-1/c_{p}}=(T_{0}+\epsilon T_{1}+\cdots ){\left(p_{0}+\epsilon p_{1}+\cdots \right)}^{-1/c_{p}},$$

which gives

$$\ln(T)=\epsilon \left(\frac{T_{1}}{T_{0}}-\frac{1}{c_{p}}\;\frac{p_{1}}{p_{0}}\right)+o(\epsilon ),$$

and then invoking (4.3)–(4.4) we obtain, at this order

$$\frac{1}{\epsilon }\;\frac{\partial \ln(T)}{\partial z}=\frac{\partial }{\partial z}\left(\frac{T_{1}}{T_{0}}-\frac{1}{c_{p}}\;\frac{p_{1}}{p_{0}}\right)=\frac{\partial }{\partial z}\left(\frac{q_{0}+A_{1}}{c_{p}T_{0}}\right)=\frac{1}{c_{p}}\;\frac{\partial^{2}\Pi_{1}}{\partial z^{2}}=\frac{1}{gc_{p}}\;\frac{\partial^{2}F_{1}}{\partial z^{2}}.$$

Therefore, we may identify

4.8 $$N^{2}=\frac{1}{c_{p}}\;\frac{\partial^{2}F_{1}}{\partial z^{2}}.$$

It is usual to assume that *N*^2^ is a constant (see [19]), in which case the general explicit solution of equation (4.7) follows directly, the necessary and sufficient condition for stability being *N*^2^ > 0. The assumption of constant *N*^2^ is clearly an oversimplification. While an explicit, general formula for the solution of equation (4.7), for *N*^2^ varying in the vertical direction, is not available, the comparison method shows that stability holds if *N*^2^ > 0 (and *N* = 0 implies neutral stability, while instability corresponds to *N*^2^ < 0); see [21].

Using (4.5), equations (3.1) and (3.2) become

4.9 $$\rho_{0}\;\frac{\partial u_{0}}{\partial t}-2\rho_{0}v_{0}\sin\theta =-\frac{\rho_{0}}{\cos\theta }\;\frac{\partial F_{1}}{\partial \phi }+\frac{1}{R_{e}}\;\frac{\partial }{\partial z}\left(m\;\frac{\partial u_{0}}{\partial z}\right)$$

and

4.10 $$\rho_{0}\;\frac{\partial v_{0}}{\partial t}+2\rho_{0}u_{0}\sin\theta =-\rho_{0}\;\frac{\partial F_{1}}{\partial \theta }+\frac{\rho_{0}\sin\theta \cos\theta }{g}\;\frac{\partial F_{1}}{\partial z}+\frac{1}{R_{e}}\;\frac{\partial }{\partial z}\left(m\;\frac{\partial v_{0}}{\partial z}\right),$$

respectively, together with

4.11 $$\frac{\partial }{\partial \phi }\;(\rho_{0}u_{0})+\frac{\partial }{\partial \theta }\;(\rho_{0}v_{0}\cos\theta )+\frac{\partial }{\partial z}\;(\rho_{0}w_{0}\cos\theta )=\frac{\rho_{0}\cos\theta }{g}\left(\frac{\partial^{2}F_{1}}{\partial z\partial t}-\frac{c_{p}-1}{c_{p}}\;\frac{g}{T_{0}}\;\frac{\partial F_{1}}{\partial t}\right),$$

from equation (3.4). Moreover, because the background state is a function of

$$\zeta =gz-\frac{1}{2}\;\cos^{2}\theta$$

only, it is useful to transform from (*φ*, *θ*, *z*, *t*) coordinates to (*φ*, *θ*, *ζ*, *t*); equations (4.9)–(4.11) then become

4.12 $$\begin{array}{rl} & \rho_{0}\;\frac{\partial u_{0}}{\partial t}-2\rho_{0}v_{0}\sin\theta =-\frac{\rho_{0}}{\cos\theta }\;\frac{\partial F_{1}}{\partial \phi }+\frac{g^{2}}{R_{e}}\;\frac{\partial }{\partial \zeta }\left(M\;\frac{\partial u_{0}}{\partial \zeta }\right), \end{array}$$

4.13 $$\begin{array}{rl} & \rho_{0}\;\frac{\partial v_{0}}{\partial t}+2\rho_{0}u_{0}\sin\theta =-\rho_{0}\;\frac{\partial F_{1}}{\partial \theta }+\frac{g^{2}}{R_{e}}\;\frac{\partial }{\partial \zeta }\left(M\;\frac{\partial v_{0}}{\partial \zeta }\right) \end{array}$$

4.14 $$\begin{array}{rl} \;\text{and}\; & \frac{\partial }{\partial \phi }\;(\rho_{0}u_{0})+\frac{\partial }{\partial \theta }\;(\rho_{0}v_{0}\cos\theta )+\frac{\partial }{\partial \zeta }\;(\rho_{0}v_{0}\sin\theta \cos^{2}\theta +g\rho_{0}w_{0}\cos\theta )=\cos\theta \;\frac{\partial^{2}(\rho_{0}F_{1})}{\partial \zeta \partial t},\; \end{array}$$

respectively, where *m*(*z*) ≡ *M*(*ζ* + (1/2) cos ^2^*θ*). This completes the development of the system that we plan to discuss here, namely, equations (4.12)–(4.14), in conjunction with (4.2) and (4.4).

## 5. Solutions of the time-dependent problem

We now turn to the main thrust of this presentation: the construction of some explicit solutions, all derived from our overarching set of general equations that arise at O(ε). In particular, we examine certain types of wave-like motion; among the plethora of unsteady motions, this is a reasonable testing ground for the applicability of our equations. Although we strongly advocate an investigation, using numerical techniques, of the complete system (4.2), (4.4) and (4.12)–(4.14), for realistic forcing functions and flow properties, e.g. suitable, variable eddy viscosity, our aim here is to produce analytical results. This will, we believe, both confirm and explain some familiar theories (but now placed in a wider context, both in terms of minimal simplifying assumptions and precise approximations and errors) and also generate new observations about the large-scale structures of the moving atmosphere. There are clearly two main avenues to explore: either assume viscous flow or take the inviscid limit; the former produces closed-form solutions only in special cases (e.g. *M* = constant), but the latter offers many possibilities. Indeed, because of the very large Reynolds number (see §2), it might be argued that, in any event, the role of viscosity can be ignored. So we look, firstly, at some inviscid flows. A comment about viscous flows will be presented in §7.

The inviscid limit of equations (4.12)–(4.14) can be written as

5.1 $$\begin{array}{rl} & \frac{\partial U}{\partial t}-2V\sin\theta =-\frac{1}{\cos\theta }\;\frac{\partial F}{\partial \phi }, \end{array}$$

5.2 $$\begin{array}{rl} & \frac{\partial V}{\partial t}+2U\sin\theta =-\frac{\partial F}{\partial \theta } \end{array}$$

5.3 $$\begin{array}{rl} \;\text{and}\; & \frac{\partial U}{\partial \phi }+\frac{\partial }{\partial \theta }\;(V\cos\theta )+\frac{\partial }{\partial \zeta }\;(V\sin\theta \cos^{2}\theta +gW\cos\theta )=\cos\theta \;\frac{\partial^{2}F}{\partial \zeta \partial t}, \end{array}$$

where we have introduced

5.4 $$\rho_{0}(u_{0},v_{0},w_{0},F_{1})=(U,V,W,F),$$

and so the explicit dependence on the background state is suppressed. This system provides a complete description of the (inviscid) velocity field at this order, for a given forcing *F*; this *F* can, in turn, be used to produce the perturbation to the thermodynamic state (given by *p*_1_, *ρ*_1_, *T*_1_), together with the identification of any required heat sources. It is of some significance that equations (5.1) and (5.2) take a very familiar form (see [19]), but the forcing here drives the leading-order velocity field, not a perturbation of it about some uniform state. On the other hand, if we assume that *W* = 0 (which is not a consistent choice within the thin-shell approximation), then equation (5.3), combined with the other two, produces a necessary constraint on *F* in order for such a solution to exist: so, we must expect that we can allow only very special heat sources to drive the atmospheric flow, in this case. We are now in a position to explore, in some detail, what equations (5.1)–(5.3) tell us about unsteady motions in the atmosphere: we outline the type of results that can be obtained by presenting a few examples, together with some general observations and a case study.

### (a) Solution harmonic in time

The simplest solution to seek is one in which all the variables are proportional to *e*^−i*ωt*^, for some real constant *ω*; so we write

$$(U,V,W,F)=(\hat{U},\hat{V},\hat{W},\hat{F})\;{\text{e}}^{-\text{i}\omega t},$$

where each of the ‘hatted’ functions depends on (*φ*, *θ*, *ζ*). From equations (5.1) and (5.2), we find directly that

5.5 $$\hat{U}=\frac{1}{\omega^{2}-4\sin^{2}\theta }\;\left\{2\sin\theta \;\frac{\partial \hat{F}}{\partial \theta }-\text{i}\;\frac{\omega }{\cos\theta }\;\frac{\partial \hat{F}}{\partial \phi }\right\}$$

and

5.6 $$\hat{V}=\frac{1}{\omega^{2}-4\sin^{2}\theta }\left\{-\text{i}\omega \;\frac{\partial \hat{F}}{\partial \theta }-2\tan\theta \;\frac{\partial \hat{F}}{\partial \phi }\right\}.$$

It should be noted that, if this horizontal velocity field is to be defined for $-\frac{\pi }{2}<\theta <\frac{\pi }{2}$ (so avoiding the Poles; see below), we must have |*ω*| > 2. We now use (5.5) and (5.6) in (5.3), and so obtain the equation relating $\hat{W}$ and $\hat{F}$:

5.7 $$\begin{array}{rl} & g\cos\theta \;\frac{\partial \hat{W}}{\partial \zeta }+\frac{1}{\omega^{2}-4\sin^{2}\theta }\;\frac{\partial }{\partial \phi }\left(2\sin\theta \;\frac{\partial \hat{F}}{\partial \theta }-\text{i}\;\frac{\omega }{\cos\theta }\;\frac{\partial \hat{F}}{\partial \phi }\right)\\ & \;-\frac{\partial }{\partial \theta }\left\{\frac{1}{\omega^{2}-4\sin^{2}\theta }\left(\text{i}\;\omega \cos\theta \;\frac{\partial \hat{F}}{\partial \theta }+2\sin\theta \;\frac{\partial \hat{F}}{\partial \phi }\right)\right\}\\ & \;-\frac{\sin\theta \cos\theta }{\omega^{2}-4\sin^{2}\theta }\;\frac{\partial }{\partial \zeta }\left(\text{i}\;\omega \cos\theta \;\frac{\partial \hat{F}}{\partial \theta }+2\sin\theta \;\frac{\partial \hat{F}}{\partial \phi }\right)=-\text{i}\;\omega \cos\theta \;\frac{\partial \hat{F}}{\partial \zeta }. \end{array}$$

Since $\hat{W}=0$ at ground level, from equation (5.7) we may determine the vertical velocity component, $w_{0}=\hat{W}/\rho_{0}$, given the forcing function $\hat{F}$ (which, in turn, produces the horizontal velocity components and the perturbation to the thermodynamic state, and can be used to identify the associated heat sources). Note that the neighbourhood of the Poles must be avoided, because this is where $\hat{W}$ is undefined, according to equation (5.7). To be more precise, we cannot allow *θ* to be in an O(ε)-neighbourhood of the Poles, for general $\hat{F}$, because the asymptotic expansions will not then be valid. Of course, the choice of $\hat{F}$ may include the requirement that $\hat{W}$ remains bounded—a reasonable condition on physical grounds—close to the Poles.

We can investigate a little further: equation (5.7) admits solutions for $\hat{F}$ harmonic in *φ* and *ζ* (but not in *θ*); we set

$$\hat{F}=\bar{F}(\theta )\;{\text{e}}^{\text{i}(k\phi +l\zeta )},$$

where *k* and *l* are constants (and we are interested in real *k*, so that we have a component of propagation in the azimuthal direction, but *l* may be complex-valued); we do not impose the same structure on $\hat{W}$ since it would amount to $\hat{W}\equiv 0$, given that $\hat{W}=0$ at ground level. The resulting equation relating $\bar{F}$ and $\hat{W}$ is

5.8 $$\begin{array}{rl} & \frac{{\text{d}}^{2}\bar{F}}{\text{d}\theta^{2}}+\left\{\left(\text{i}l+\frac{8}{\omega^{2}-4\sin^{2}\theta }\right)\sin\theta \cos\theta -\tan\theta \right\}\;\frac{\text{d}\bar{F}}{\text{d}\theta }\\ & \;+\left\{-\frac{k^{2}}{\cos^{2}\theta }+2\;\frac{k}{\omega }\;+2\text{i}\;\frac{lk}{\omega }\;\sin^{2}\theta -\text{i}l(\omega^{2}-4\sin^{2}\theta )+16\;\frac{k}{\omega }\;\frac{\sin^{2}\theta }{\omega^{2}-4\sin^{2}\theta }\right\}\bar{F}\\ & \;+\frac{\text{i}g}{\omega }\;(\omega^{2}-4\sin^{2}\theta )\;\frac{\partial \hat{W}}{\partial \zeta }=0. \end{array}$$

#### (i) Reappraisal of the classical dispersion relation

While the dependence on *θ* in equation (5.8) makes any general further development challenging, we can be confident that we have the correct representation of the behaviour in the meridional direction. It is therefore this equation that should be examined if we require a consistent representation in the neighbourhood of particular *θ*; this we now do by seeking a harmonic solution close to *θ* = *θ*_0_. To accomplish this, we evaluate all the coefficients in equation (5.8) on *θ* = *θ*_0_, and then seek a zonally harmonic solution:

$$\bar{F}=F_{0}\;{\text{e}}^{\text{i}m(\theta -\theta_{0})},$$

where *m* is a constant, not necessarily real. The resulting ‘dispersion’ relation becomes

5.9 $$\begin{array}{rl} & F_{0}\{2kl\sin^{2}\theta_{0}\cos\theta_{0}+\frac{8\omega m\sin\theta_{0}\cos^{2}\theta_{0}}{\omega^{2}-4\sin^{2}\theta_{0}}-\omega m\sin\theta_{0}-\omega l(\omega^{2}-4\sin^{2}\theta_{0})\cos\theta_{0}\\ & \;+\text{i}(\frac{\omega k^{2}}{\cos\theta_{0}}+\omega m^{2}\cos\theta_{0}+\omega ml\sin\theta_{0}\cos^{2}\theta_{0}-2k\cos\theta_{0}-\frac{16k\sin^{2}\theta_{0}\cos\theta_{0}}{\omega^{2}-4\sin^{2}\theta_{0}})\}\\ & \;+g\cos\theta_{0}\;(\omega^{2}-4\sin^{2}\theta_{0})\;\frac{\partial \hat{W}}{\partial \zeta }=0. \end{array}$$

We should note that, if we seek such a solution, harmonic in (*φ*, *ζ*, *t*) and locally harmonic in *θ*, which is then evaluated for zero heat input, i.e. *F*_0_ = 0, then there is no motion at this order. Equation (5.9) is not a dispersion relation in any conventional sense, unless some very special assumptions are made about $\partial \hat{W}/\partial \zeta /F_{0}$; rather, this equation determines $\hat{W}$, given *F*_0_ and all the wavenumbers and the frequency. However, our approach enables us to explain how a familiar result can be recovered. In equation (5.9), we ignore all the terms that are generated by the existence of a background state or that arise from the spherical geometry other than the term involving $\partial \hat{W}/\partial \zeta$; in addition, we assume that there is no contribution from a heat source, which then leaves us with

$$F_{0}\left\{\frac{\omega k^{2}}{\cos\theta_{0}}+\omega m^{2}\cos\theta_{0}\right\}-\text{i}g\cos\theta_{0}\;(\omega^{2}-4\sin^{2}\theta_{0})\;\frac{\partial \hat{W}}{\partial \zeta }=0.$$

Even though *θ*_0_ may take any value, we must insist that cos*θ*_0_ = 1 in this context; finally, we also assume that there is an auxiliary condition (which is not available at this order), which gives

$$\frac{\frac{\partial \hat{W}}{\partial \zeta }}{F_{0}}=-\frac{\omega l}{gN^{2}}$$

and so

$$\omega \{l^{2}\omega^{2}-[(k^{2}+m^{2})N^{2}]+4l^{2}\sin^{2}\theta_{0}]\}=0,$$

the standard result involving the Brunt–Väisälä frequency, *N*; see, for example, [19]. In other words, the familiar result has no bearing on, or relevance to, the movement of the atmosphere: it cannot be recovered in any systematic analysis of the governing equations. (The errors arise as follows: (i) it is inconsistent to replace cos*θ* by 1 in the evaluation of a term such as (∂/∂*θ*){cos*θ*(∂/∂*θ*) (*v*cos*θ*)}, (ii) there is no background state and (iii) there is no equation available, at this order, to allow the introduction of *N*^2^.)

We now return to the general time-harmonic version of our equations, (5.5)–(5.7), and seek solutions that are exponential in *φ* and *ζ*:

5.10 $$\hat{F}(\phi ,\theta ,\zeta )={\text{e}}^{\alpha \phi +\gamma \zeta }\;f(\theta ),$$

where *α* and *γ* are constants (which may be chosen to be real or complex, as appropriate, in specific cases). Thus equations (5.5) and (5.6) become

5.11 $$\hat{U}=\frac{1}{\omega^{2}-4\sin^{2}\theta }\left(2\sin\theta \;\frac{\text{d}f}{\text{d}\theta }-\text{i}\;\frac{\alpha \omega }{\cos\theta }\;f\right){\text{e}}^{\alpha \phi +\gamma \zeta }$$

and

5.12 $$\hat{V}=-\frac{1}{\omega^{2}-4\sin^{2}\theta }\left(\text{i}\omega \;\frac{\text{d}f}{\text{d}\theta }+2\alpha \tan\theta \;f\right){\text{e}}^{\alpha \phi +\gamma \zeta },$$

respectively; correspondingly, equation (5.7), after an integration in *ζ* and imposing the boundary condition $\hat{W}=0$ at the bottom of the atmosphere (i.e. on $\zeta =-\frac{1}{2}\;\cos^{2}\theta$), becomes

5.13 $$\begin{array}{rl} \hat{W} & =\frac{1}{g\gamma \cos\theta }\;\{\frac{\sin(2\theta )}{2(\omega^{2}-4\sin^{2}\theta )}(\text{i}\omega \gamma \cos\theta \;\frac{\text{d}f}{\text{d}\theta }+2\alpha \gamma f\sin\theta )\\ & -\frac{1}{\omega^{2}-4\sin^{2}\theta }\;(2\alpha \sin\theta \;\frac{\text{d}f}{\text{d}\theta }-\frac{\text{i}\omega \alpha^{2}}{\cos\theta }\;f)-\text{i}\omega \gamma f\cos\theta \\ & +\frac{\text{d}}{\text{d}\theta }\;[\frac{1}{\omega^{2}-4\sin^{2}\theta }\;(\text{i}\omega \cos\theta \;\frac{\text{d}f}{\text{d}\theta }+2\alpha f\sin\theta )]\}({\text{e}}^{\gamma \zeta }-{\text{e}}^{-\frac{1}{2}\;\gamma \cos^{2}\theta }){\text{e}}^{\alpha \phi }. \end{array}$$

#### (ii) Standing waves

As a simple illustration of time-harmonic solutions, we consider flows oscillating in the region of the tropics (so we take $|\theta |\leq 23^{\circ }$). Let $\hat{W}=0$ (so there is no motion in the vertical direction and then the bottom boundary condition is automatically satisfied), with a heat forcing $\hat{F}$ that depends on only *θ* (so we take *α* = *γ* = 0 in (5.10)). With these conditions used in equation (5.13), we find that

$$\frac{\text{d}f}{\text{d}\theta }=\frac{\omega^{2}-4\sin^{2}\theta }{\cos\theta }\;f_{0}{\text{e}}^{\text{i}\theta_{0}},$$

for some real constants *f*_0_ > 0 and *θ*_0_ ∈ [0, 2*π*), and then, using (5.11) and (5.12) in (5.5) and (5.6), respectively, we obtain

$$\hat{U}=2f_{0}\;{\text{e}}^{\text{i}(\theta_{0}-\omega t)}\tan\theta ,\;\hat{V}=\frac{\omega f_{0}}{\cos\theta }\;{\text{e}}^{\text{i}(-(\pi /2)+\theta_{0}-\omega t)}\;\text{and}\;\hat{W}=0.$$

This solution represents a standing wave, i.e. oscillating in time but not propagating. We note that, even though the expressions derived above exhibit no variation in the vertical direction, (5.4) shows that there is a vertical structure, by virtue of *ρ*_0_, in the horizontal velocity field (*u*_0_, *v*_0_).

#### (iii) Modulated travelling wave

**Figure 2.**
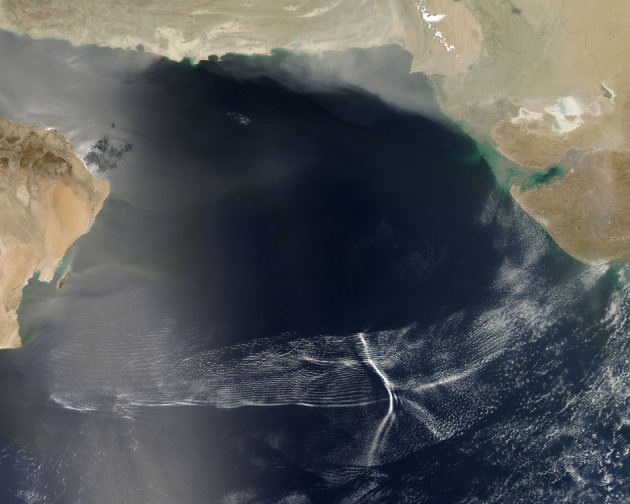
Natural-colour photograph of atmospheric waves spanning the Arabian Sea from Oman to India, taken on 8 May 2007, by NASA’s Acqua satellite (Image Credit: NASA). A low-level, high-speed jet stream (the Findlater Jet; see figure 3) cuts across the centre of the wave pattern, creating a distinctive parabolic-shaped cloud; clouds of dust from Iran, Pakistan and Oman are also visible. The air flow roughens the surface of the ocean: bands of light and dark water that mimic the wave pattern are visibile near the shore of Oman, where the sunlight reflected off the water is picked up directly by the satellite’s sensor (with a rough water surface dispersing light and thus creating the dark bands, while calm water is brighter). (Online version in colour.)

Waves propagating towards the Equator (for example, as shown in figure 2) can be obtained from our system (5.11)–(5.13) by setting *α* = 0 (so there is no component of propagation in the azimuthal direction) and then by choosing a suitable *f*(*θ*). A simple choice, which produces no motion on the Equator, is

$$\frac{1}{\omega^{2}-4\sin^{2}\theta }\;\frac{\text{d}f}{\text{d}\theta }=A{\text{e}}^{\text{i}\lambda \theta }\sin\theta ,$$

where *A* is an arbitrary (complex) constant and *λ* some real constant. Then, from (5.11) and (5.12), and reinstating the harmonic time-dependence, we obtain a modulated wave motion described by

$$U=2A{\text{e}}^{\text{i}(\lambda \theta -\omega t)+\gamma \zeta }\sin^{2}\theta \;\text{and}\;V=-\text{i}\omega A{\text{e}}^{\text{i}(\lambda \theta -\omega t)+\gamma \zeta }\sin\theta ,$$

where *γ* may be chosen to be complex-valued (to produce an exponential behaviour with height, together with a tilt in the direction of propagation). Indeed, we could simplify further by imposing the condition |*γ*| → 0, which produces the simpler solution

$$U=2A{\text{e}}^{\text{i}(\lambda \theta -\omega t)}\sin^{2}\theta \;\text{and}\;V=-\text{i}\omega A{\text{e}}^{\text{i}(\lambda \theta -\omega t)}\sin\theta$$

and

$$W=\frac{\text{i}\omega A}{g}\;\frac{\zeta +\frac{1}{2}\;\cos^{2}\theta }{\cos\theta }\left(\cos(2\theta )+\frac{1}{2}\;\text{i}\lambda \sin(2\theta )\right){\text{e}}^{\text{i}(\lambda \theta -\omega t)}.$$

This solution, we observe, describes *W* varying linearly with height, but our development makes clear that many other choices are available, for which suitable heat sources can be identified.

**Figure 3.**
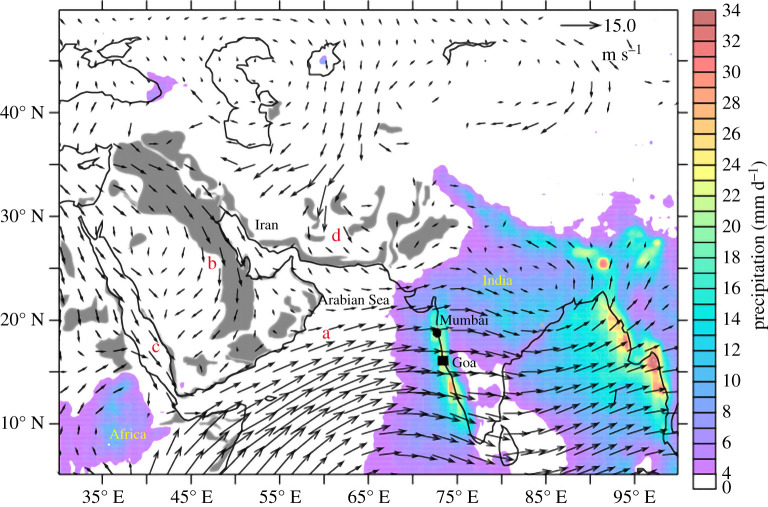
Climatology (2003–2014) of the major winds at the 850 hPa level (about 1.5 km above sea level) over the northern Indian Ocean during the summer monsoon period, with the major dust-source regions shown in grey: (*a*) the Findlater Jet (see [24]); (*b*) the Shamal winds; (*c*) the Red Sea Winds; (*d*) the Levar Winds. Reproduced from [25], CC by Springer Nature. (Online version in colour.)

The above analysis demonstrates that we can reasonably expect to match what is observed. Of course, the identification of flow structures and processes from the various available satellite data is rather subjective [22], but such exercises nevertheless provide major sources of insight. In the case of the wave pattern visible in figure 2, it is observed, during May, that the surface temperature of the north Indian Ocean becomes the highest among the world’s ocean surface temperatures, often in excess of $28^{\circ }$C over the Arabian Sea, where evaporation largely exceeds precipitation; see [23]. The sea-surface temperature is comparable with the daily maximal land temperature in the coastal areas adjacent to the Arabian Sea, with a significant drop in temperature at night over the land. These changes in the temperature over space and time can give rise to wave patterns of the type shown in figure 2. In addition, there is a strong eastward flow near the Equator, as shown in figure 3. In an attempt to recover this general flow pattern, we make a different ansatz (but still invoking |*γ*| → 0 for simplicity), which produces an appropriate solution in equatorial regions: set *α* = *ia* where *a* is a real constant, and choose *f*(*θ*) = *b*, where *b* is a complex constant. This results in the solution

$$U=\frac{\omega ab}{(\omega^{2}-4\sin^{2}\theta )\cos\theta }\;{\text{e}}^{\text{i}(a\phi -\omega t)}\;\text{and}\;V=-\frac{2\text{i}ab\tan\theta }{\omega^{2}-4\sin^{2}\theta }\;{\text{e}}^{\text{i}(a\phi -\omega t)},$$

which describes a wave propagating in the azimuthal direction (with *V* = 0 and *U* ≠ 0 along the Equator), and

$$W=\frac{\text{i}ab(\zeta +\frac{1}{2}\;\cos^{2}\theta )}{g{\left(\omega^{2}-4\sin^{2}\theta \right)}^{2}\cos^{2}\theta }\;[2(\omega^{2}+4\sin^{2}\theta )\cos^{2}\theta -\omega a(\omega^{2}-4\sin^{2}\theta )]\;{\text{e}}^{\text{i}(a\phi -\omega t)}.$$

These two examples demonstrate that our single formulation can capture different modes and types of wave propagation in the atmosphere, avoiding the need for *ad hoc* modelling.

### (b) Equatorially trapped waves

Due to the nature of their energy sources, as well as the small Coriolis forcing, large-scale equatorial atmospheric flows present specific structural features. Synoptic-scale disturbances of the atmosphere outside the tropics are mainly driven by horizontal temperature gradients, the primary energy source being the potential energy associated with the latitudinal temperature gradient [19]. In the tropics, however, horizontal temperature gradients are very small, and the energy harnessed by atmospheric flows comes from diabatic heating due to latent heat release, mostly occurring in association with moist convection. The cloudiest regions on Earth (with the oceans significantly cloudier than land) are the tropics, and the higher temperatures in these regions enhance water evaporation into the air, so that the atmosphere becomes very humid. The rapid drop of temperature and pressure with altitude causes some of the water vapour in the air to condense, forming clouds (on average 19${}^{\circ }$K colder than the ground) and, if enough water condenses, the cloud droplets can become large enough to fall as rain. There is a considerable release of heat during precipitations, which alters the thermal equilibrium and triggers an adjustment process by means of fast propagating waves (with speeds of the order of 20 m s^−1^; see [26]). This local reaction of the atmospheric flow may also induce remote responses through the excitation of equatorial waves. In particular, equatorially trapped waves are related to the heat forcing that occurs over the Indonesian ‘maritime continent’, an area straddling the Equator in the middle of the warmest body of water (between the Indian and western Pacific warm pools), whose land-sea heat-capacity differences (due to the mixture of ocean and islands with long coastlines) generate the world’s largest rainfall on diurnal cycles, with enormous energy release by convective condensation. With all this in mind, we investigate how our system of equations might model some aspects of these phenomena.

We start by imposing a vanishing meridional velocity component (a reasonable assumption in equatorial regions), and then the system (5.1)–(5.3) reduces to

5.14 $$\begin{array}{rl} & \frac{\partial U}{\partial t}=-\frac{1}{\cos\theta }\;\frac{\partial F}{\partial \phi }, \end{array}$$

5.15 $$\begin{array}{rl} & 2U\sin\theta =-\frac{\partial F}{\partial \theta } \end{array}$$

5.16 $$\begin{array}{rl} \text{and}\; & \frac{\partial U}{\partial \phi }+g\cos\theta \;\frac{\partial W}{\partial \zeta }=\cos\theta \;\frac{\partial^{2}F}{\partial \zeta \partial t}. \end{array}$$

We now seek a solution generated by a heat forcing $\tilde{F}(\eta ,\theta ,\zeta )$, where *η* = *φ* − *ct*, which is azimuthally localized (that is, it vanishes for |*η* − *η*_0_| ≥ *α*, for suitable *η*_0_ and some *α* > 0, which is taken to be fairly small); furthermore, this function is to be symmetric about the Equator and describes a heat source that moves in the zonal direction at constant speed, *c*. From (5.15), we obtain

5.17 $$U(\eta ,\theta ,\zeta )=-\frac{1}{2\sin\theta }\;\frac{\partial \tilde{F}}{\partial \theta }(\eta ,\theta ,\zeta ),$$

and the meridional symmetry of $\tilde{F}$ ensures (by l’Hospital’s rule) that *U* is not singular along *θ* = 0. Combining (5.14) with (5.17) yields the differential equation

5.18 $$\frac{\partial }{\partial \theta }\left(\frac{\partial \tilde{F}}{\partial \eta }\right)=-\left(\frac{2}{c}\tan\theta \right)\frac{\partial \tilde{F}}{\partial \eta },$$

which can be integrated directly to give

$$\frac{\partial \tilde{F}}{\partial \eta }(\eta ,\theta ,\zeta )=A(\eta ,\zeta )\;{\left[\cos\theta \right]}^{2/c},$$

for some function *A* ≥ 0 (because the heat forcing decreases poleward) and such that *A*(*η*, *ζ*) = 0 for |*η* − *η*_0_| ≥ *α*. Since $\tilde{F}(\eta ,\theta ,\zeta )$ must vanish for |*η* − *η*_0_| ≥ *α*, integration gives

5.19 $$\tilde{F}(\eta ,\theta ,\zeta )=B(\eta ,\zeta )\;{\left[\cos\theta \right]}^{2/c},$$

where $B(\eta ,\zeta )=\int_{\eta_{0}-\alpha }^{\eta }A(s,\zeta )\;\text{d}s$ and then

5.20 $$\int_{\eta_{0}-\alpha }^{\eta_{0}+\alpha }A(s,\zeta )\;\text{d}s=0,$$

to ensure that *B*(*η*, *ζ*) = 0 for |*η* − *η*_0_| ≥ *α*. From (5.17), we obtain

5.21 $$U(\eta ,\theta ,\zeta )=\frac{1}{c}\;B(\eta ,\zeta )\;{\left[\cos\theta \right]}^{(2/c)-1}.$$

Since *A* ≥ 0 and the heat forcing decreases away from the Equator, from (5.19), we infer that necessarily *c* > 0. Furthermore, (5.21) demonstrates two essential properties: eastward propagation (because *c* > 0) and equatorial trapping. To gain insight into the meridional decay (i.e. equatorial trapping) of the flow, note that the non-dimensional speed *c* corresponds to $c\Omega^{\prime }d_{E}^{\prime }$ (in m s^−1^), and then *c* ≈ 23 produces a realistic value of 20 m s^−1^. For this value, (5.21) predicts a threefold reduction at 12 degrees of latitude, while at the boundary of the tropics, corresponding to a latitude of about 23 degrees, the flow attains merely 2% of its equatorial strength. On the other hand, combining (5.16) and (5.19)–(5.21) yields

5.22 $$\frac{\partial W}{\partial \zeta }=-\frac{1}{gc}\;{\left[\cos\theta \right]}^{(2/c)-2}\left\{A+c^{2}\cos^{2}\theta \;\frac{\partial A}{\partial \zeta }\right\}.$$

The fact that *W* = 0 at ground level *ζ* = −(1/2) cos ^2^*θ* rules out the possibility that ∂*W*/∂*ζ* < 0 somewhere at ground level. Since (5.20) ensures the existence of regions where *A* > 0, we conclude from (5.22) that *A* (and therefore the heat forcing) must feature variations in the vertical direction, with ∂*A*/∂*ζ* < 0 at ground level. Field data (see fig. 12 in [27]) confirms that the heat forcing typically decreases in the lower and in the upper tropical troposphere, and it increases with respect to height at mid-altitudes (roughly between 2 and 8 km).

The classical theoretical approach for describing equatorially trapped waves (see [19]) concentrates on their horizontal structure, using a shallow water model (for a fluid system of mean depth *h*_*e*_ in a motionless basic state) and the equatorial *β*-plane approximation. While the predictions made by relying on these approximations (non-dispersive, eastward propagating trapped waves) are somewhat similar to ours, there are significant differences. Firstly, approximating sin*θ* and cos*θ* by the first two terms in their Taylor expansions near *θ* = 0 leads to an exponential meridional decay rate; ours is not so severe because we have not approximated the *θ*-dependence. Secondly, the wave speed is taken to be $c=\sqrt{gh_{e}}$, i.e. that for shallow water gravity waves, although an adequate choice of *h*_*e*_ is somewhat elusive. In contrast to this, since *F* = *ρ*_0_*F*_1_, we deduce from (4.5) that the value of *F* at ground level is determined by that of the perturbation pressure, which therefore, due to (5.19), determines the speed *c*. The third aspect is more fundamental: the classical approach ignores variations of the heat forcing in the vertical direction, while the above discussion of the implications of (5.22) shows that this is an essential aspect of the dynamics. In this context, a simple example of type (5.19)–(5.20) that captures the main observed features is

5.23 $$F(\phi ,\theta ,\zeta )=a(\phi )\;H(\zeta )\;{\left[\cos\theta \right]}^{2/c},$$

where, in analogy to the considerations discussed in [28],

$$a(\phi )=\left\{ \begin{array}{ll} \cos[\frac{\pi }{2\alpha }\;(\phi -\phi_{0})], & |\phi -\phi_{0}|<\alpha ,\\ 0, & |\phi -\phi_{0}|\geq \alpha , \end{array}\right.$$

and where *H*(*ζ*) is the restriction of a cubic polynomial in *ζ* to the interval [ − (1/2), *g*], which decreases for small and large values of *ζ* and increases in the middle part of the interval (thus capturing the previously discussed typical monotonicity with height of the heat forcing throughout the tropical troposphere). Such a polynomial can be easily obtained by means of Lagrange interpolation, using field data to identify the relevant values at the top and bottom of the troposphere, and at the two critical points in-between, where the monotonicity of the heat-forcing-profile changes.

### (c) No additional constraints: general unsteady motion

The horizontal, inviscid velocity field can be obtained directly from equations (5.1) and (5.2) without imposing any additional constraints. For fixed (*φ*, *θ*, *ζ*), we write (5.1)–(5.2) as the inhomogeneous linear differential system

$$\frac{\text{d}}{\text{d}t}\;\left(\begin{array}{c} U\\ V \end{array}\right)=\left(\begin{array}{cc} 0 & 2\sin\theta \\ -2\sin\theta & 0 \end{array}\right)\left(\begin{array}{c} U\\ V \end{array}\right)+\left(\begin{array}{c} -\frac{1}{\cos\theta }\;\frac{\partial F}{\partial \phi }\\ -\frac{\partial F}{\partial \theta } \end{array}\right),$$

whose general solution is given by the variation-of-constants formula (see [29])

5.24 $$\left(\begin{array}{c} U(\phi ,\theta ,\zeta ,t)\\ V(\phi ,\theta ,\zeta ,t) \end{array}\right)={\text{e}}^{At}\left(\begin{array}{c} C(\phi ,\theta ,\zeta )\\ D(\phi ,\theta ,\zeta ) \end{array}\right)+\int_{0}^{t}{\text{e}}^{A(t-s)}\left(\begin{array}{c} -\frac{1}{\cos\theta }\;\frac{\partial F}{\partial \phi }(\phi ,\theta ,\zeta ,s)\\ -\frac{\partial F}{\partial \theta }(\phi ,\theta ,\zeta ,s) \end{array}\right)\;\text{d}s,$$

where *C* and *D* are arbitrary functions, and

$${\text{e}}^{At}=\sum_{k\geq 0}^{\infty }\frac{t^{k}}{k!}\;A^{k}\;\text{with}\;A=\left(\begin{array}{cc} 0 & 2\sin\theta \\ -2\sin\theta & 0 \end{array}\right).$$

The fundamental solution of the associated homogeneous constant-coefficient system

$$\frac{\text{d}}{\text{d}t}\;\left(\begin{array}{c} U\\ V \end{array}\right)=\left(\begin{array}{cc} 0 & 2\sin\theta \\ -2\sin\theta & 0 \end{array}\right)\left(\begin{array}{c} U\\ V \end{array}\right)$$

can be found directly as

$${\text{e}}^{At}=\left(\begin{array}{cc} \cos(2t\sin\theta ) & \sin(2t\sin\theta )\\ -\sin(2t\sin\theta ) & \cos(2t\sin\theta ) \end{array}\right),$$

and consequently

5.25 $$\begin{array}{rl} U(\phi ,\theta ,\zeta ,t) & =C(\phi ,\theta ,\zeta )\;\cos(2t\sin\theta )+D(\phi ,\theta ,\zeta )\;\sin(2t\sin\theta )\\ & \;-\int_{0}^{t}\left(\frac{\cos[2(t-s)\sin\theta ]}{\cos\theta }\;\frac{\partial F}{\partial \phi }+\sin[2(t-s)\sin\theta ]\;\frac{\partial F}{\partial \theta }\right)\text{d}s \end{array}$$

and

5.26 $$\begin{array}{rl} V(\phi ,\theta ,\zeta ,t) & =D(\phi ,\theta ,\zeta )\;\cos(2t\sin\theta )-C(\phi ,\theta ,\zeta )\;\sin(2t\sin\theta )\\ & \;+\int_{0}^{t}\left(\frac{\sin[2(t-s)\sin\theta ]}{\cos\theta }\;\frac{\partial F}{\partial \phi }-\cos[2(t-s)\sin\theta ]\;\frac{\partial F}{\partial \theta }\right)\text{d}s. \end{array}$$

Corresponding solutions for *W* can be obtained from equation (5.3). Furthermore, we see that this describes the solution that arises when the external forcing is zero (*F* ≡ 0): it is unsteady but of a very specific form. This formulation, therefore, enables the solution to be found for any given forcing, the choice of which can be guided by the available data; this is clearly an area for extensive investigation.

## 6. Case study: the African Easterly Jet/Waves

During the Northern Hemisphere summer, strong heating of the Saharan region in North Africa, and the relatively cool and moist air to its south (in the Gulf of Guinea), creates a situation in which the usual north–south horizontal temperature gradient is reversed in the lower troposphere above the Sahel region, while in the upper troposphere the insolation-induced horizontal decrease of temperature with increasing latitude persists. Observations show the appearance of a strong westward jet, called the African Easterly Jet (AEJ), whose core on the western coast of Africa is near 15${}^{\circ }$ N, at a height of about 4 km (where the reversal of the temperature gradient in the middle troposphere occurs); see [30,31]. Denoting by a bracket the temporal mean (recall that 3$\frac{1}{2}$ h corresponds to a unit interval for the non-dimensional variable *t*), in the time-periodic setting, we see that (5.1)–(5.3) yield the steady system

$$\begin{array}{rl} & -2\left\langle V\right\rangle \sin\theta =-\frac{1}{\cos\theta }\;\frac{\partial \left\langle F\right\rangle }{\partial \phi },\\ & 2\left\langle U\right\rangle \sin\theta =-\frac{\partial \left\langle F\right\rangle }{\partial \theta }\\ \;\text{and}\; & \frac{\partial \left\langle U\right\rangle }{\partial \phi }+\frac{\partial \left\langle V\right\rangle }{\partial \theta }\;\cos\theta -\left\langle V\right\rangle \sin\theta +\frac{\partial \left\langle V\right\rangle }{\partial \zeta }\;\sin\theta \cos^{2}\theta +g\;\frac{\partial \left\langle W\right\rangle }{\partial \zeta }\;\cos\theta =0, \end{array}$$

so that

6.1 $$\begin{array}{rl} & \left\langle U\right\rangle =-\frac{1}{2\sin\theta }\;\frac{\partial \left\langle F\right\rangle }{\partial \theta }, \end{array}$$

6.2 $$\begin{array}{rl} & \left\langle V\right\rangle =\;\frac{1}{2\sin\theta \cos\theta }\;\frac{\partial \left\langle F\right\rangle }{\partial \phi } \end{array}$$

6.3 $$\begin{array}{rl} \;\text{and}\; & \frac{\partial \left\langle W\right\rangle }{\partial \zeta }=\frac{1}{2g}\left(\frac{1}{\sin^{2}\theta }\;\frac{\partial \left\langle F\right\rangle }{\partial \phi }-\frac{\partial^{2}\left\langle F\right\rangle }{\partial \phi \partial \zeta }\right). \end{array}$$

Since *W* = 0 at the ground level (*ζ* = −(1/2) cos ^2^*θ*), integration of equation (6.3) gives

6.4 $$\left\langle W\right\rangle (\phi ,\theta ,\zeta )=-\frac{1}{2g}\;\frac{\partial \left\langle F\right\rangle }{\partial \phi }{|}_{\zeta =-(1/2)\;\cos^{2}\theta }^{\zeta }+\frac{1}{2g\sin^{2}\theta }\int_{-(1/2)\;\cos^{2}\theta }^{\zeta }\frac{\partial \left\langle F\right\rangle }{\partial \phi }(\phi ,\theta ,s)\;\text{d}s.$$

The relations (6.1)–(6.3) enable us to infer the basic dynamics of the AEJ from the observed behaviour of the heat forcing *F*. The heat-flux contribution from clouds is negligible, the main source being the ground-level temperature gradient, distributed in the lower troposphere by dry convection and diffusion (rather than by condensational heating); see the discussion in [30]. Going north from the Guinean coast towards the Sahara, in the region from 10${}^{\circ }$ N to 20${}^{\circ }$ N, the surface temperature increases gradually by about 10${}^{\circ }$ K (see the data in [30]). Thus $\frac{\partial \left\langle F\right\rangle }{\partial \theta }>0$ and (6.1) predicts a westward zonal flow component for the AEJ. On the other hand, the surface temperature in the lower-troposphere Sahel region between 15${}^{\circ }$ W and 10${}^{\circ }$ E increases (weakly) eastwards (see the data in [30]). The fact that ∂〈*F*〉/∂*φ* > 0 in the lower troposphere yields, from (6.2), that the meridional flow velocity of the AEJ is northward. Field data (see [30]) also show that near 15${}^{\circ }$ N, between 15${}^{\circ }$ W and 10${}^{\circ }$ E, the positive longitudinal gradient of the temperature increases with height near the ground and then starts to decrease towards the mid-troposphere altitude, where it reverses sign: the classic Hadley circulation coexists with a second but shallower overturning circulation in the lower part of the troposphere (see [32]). Due to (6.2), at a fixed latitude *θ*, we can track the variation of ∂〈*F*〉/∂*φ* with height by the corresponding variation of the meridional velocity component, *V*. Field data for the AEJ (see [30,33]) show that *V* increases linearly with height in the lower third of the troposphere, from about 1–2 m s^−1^ near the ground, to a maximum of about 6–7 m s^−1^ at an altitude of about 4 km. Given that one non-dimensional speed unit corresponds to $\Omega^{\prime }H^{\prime }∼1.16\;{\text{m s}}^{-1}$, this means that the vertical slope of ∂〈*F*〉/∂*φ* near the ground is less than 15. Since $\sin(15^{\circ })∼0.258$, we see that, for the AEJ, 1/sin ^2^*θ* exceeds the vertical slope of ∂〈*F*〉/∂*φ* in the lower third of the troposphere. Consequently, (6.3) confirms that uplifting (*W* > 0) occurs near the ground. We have presented the main qualitative features of the AEJ, but detailed data about the temperature (which is readily available, but its use is outside the scope of this initial investigation) would provide the basis for a quantitative analysis, producing a more comprehensive description.

**Figure 4.**
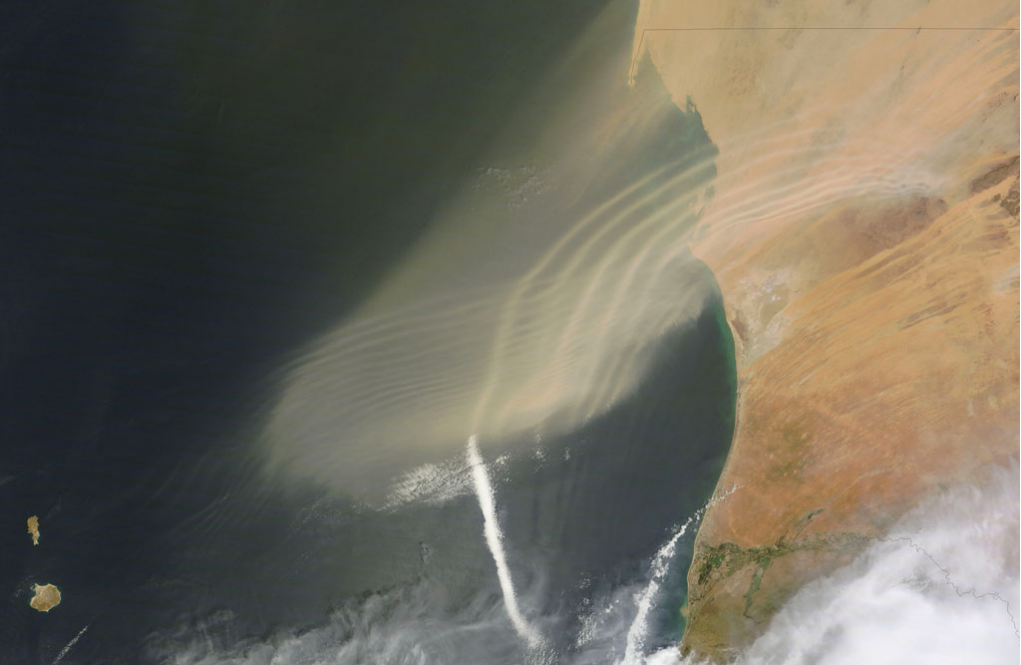
Natural-colour photograph of the north-western coastal region of Africa, taken on 23 September 2011, by NASA’s Terra satellite (Image Credit: NASA). This photograph captures two atmospheric waves made visible by dust (sand) blowing from the Sahara Desert, these waves being along the tracks of the two predominant air flows in this region. The waves with longer wavelength (of about 20 km) propagate at about 10 m s^−1^ in the southwest-northeast direction of the African Easterly Jet (AEJ)—a permanent air flow in the lower troposphere, thermally induced by the contrast between the warm Sahara Desert area to the north and the cool Gulf of Guinea to the south—upward to 6 km above the ground, with the core at about 3 km above the ground (where the vertically sheared AEJ is most intense; see [35]). The shorter waves propagating northward at about 3 m s^−1^ arise as perturbations of an ageostrophic meridional circulation in the upper troposphere, mainly in the region of 8–12 km above ground; see [36]. Note that the westward travelling African Easterly Waves, arising as lower-tropospheric three-dimensional synoptic-scale disturbances of the AEJ (see [37]), with a maximal speed of about 11 m s^−1^ (attained around 4 km altitude) and wavelengths of about 2000 km, are not visible in the photograph. These more intricate wave patterns are identified by using theoretical predictions to interpret field data, for example, by performing a wavenumber-frequency spectrum analysis to isolate statistically significant spectral peaks that correspond to the available dispersion relations (see [38]). (Online version in colour.)

African easterly waves (AEWs) are westward moving oscillatory disturbances of the AEJ, initiated by mesoscale convective systems over Central Africa and propagating in the lower troposphere; see [34]. The wave periods are within the range of 2–9 days and the wavelengths are about 2000 km, with maximal speeds of about 11 m s^−1^. A model for these AEWs can be obtained from the system (5.1)–(5.3) by constructing time-dependent perturbations $\left(\tilde{U},\tilde{V},\tilde{W}\right)$ of the AEJ mean flow, (〈*U*〉, 〈*V*〉, 〈*W*〉), driven by a suitable time-dependent heat forcing $\tilde{F}$, a perturbation of the mean heat forcing 〈*F*〉. With the mean flow determined by (6.1)–(6.3), the waves are therefore solutions of the system

$$\begin{array}{rl} & \frac{\partial \tilde{U}}{\partial t}-2\tilde{V}\sin\theta =-\frac{1}{\cos\theta }\;\frac{\partial \tilde{F}}{\partial \phi },\\ & \frac{\partial \tilde{V}}{\partial t}+2\tilde{U}\sin\theta =-\frac{\partial \tilde{F}}{\partial \theta }\\ \;\text{and}\; & \frac{\partial \tilde{U}}{\partial \phi }+\frac{\partial }{\partial \theta }\;(\tilde{V}\cos\theta )+\frac{\partial }{\partial \zeta }\;(\tilde{V}\sin\theta \cos^{2}\theta +g\tilde{W}\cos\theta )=\cos\theta \;\frac{\partial^{2}\tilde{F}}{\partial \zeta \partial t}, \end{array}$$

whose general solution was described in §5*c*. Waves with shorther wavelengths than the AEWs are also observed (figure 4); the above approach also applies in this case.

## 7. Viscous flow

We return to the original set of equations that define the velocity field at this order, namely equations (4.12)–(4.14). An analysis of this system is essential if viscous effects are thought to play a role in the atmospheric flows of interest, which is certainly the case within the atmospheric boundary layer (see the discussion in [39]). The general procedure is exactly as before: solve the first two for the horizontal components, and then the third gives the vertical component (or provides an auxiliary condition, if we choose to set *w*_0_ = 0). The simplest way to proceed, as was done in [3], is to introduce the complex velocity:

$$u_{0}+\text{i}v_{0}=Z_{0}(\phi ,\theta ,\zeta \;t);$$

equations (4.12) and (4.13) then give

7.1 $$\frac{\partial Z_{0}}{\partial t}+2\text{i}Z_{0}\sin\theta =\frac{g^{2}}{\rho_{0}R_{e}}\;\frac{\partial }{\partial \zeta }\left(M\;\frac{\partial Z_{0}}{\partial \zeta }\right)-\frac{1}{\rho_{0}}\left(\frac{1}{\cos\theta }\;\frac{\partial }{\partial \phi }+\text{i}\;\frac{\partial }{\partial \theta }\right)(\rho_{0}F_{1}).$$

This can be rewritten in a slightly more convenient form by setting

$$Z_{0}(\phi ,\theta ,\zeta \;t)={\text{e}}^{-2\text{i}t\sin\theta }Y_{0}(\phi ,\theta ,\zeta \;t),$$

to obtain

7.2 $$\frac{\partial Y_{0}}{\partial t}-\frac{g^{2}}{\rho_{0}R_{e}}\;\frac{\partial }{\partial \zeta }\;\left(M\;\frac{\partial Y_{0}}{\partial \zeta }\right)=-\frac{{\text{e}}^{-2\text{i}t\sin\theta }}{\rho_{0}}\;\left(\frac{1}{\cos\theta }\;\frac{\partial }{\partial \phi }+\text{i}\;\frac{\partial }{\partial \theta }\right)(\rho_{0}F_{1}).$$

By means of a Liouville substitution, equation (7.2) can be transformed into a (complex-valued) Bessel equation (after the removal of a time-dependent factor), whose general solution can be expressed in terms of Bessel functions of the first kind (at least, for some reasonable choices of *M*); see [3]. It is clear that the development of these solutions is quite involved but readily accessible, and the results should provide important insights into these flows; this is another area that is left for future investigation.

## 8. Discussion

The ideas developed in this paper have shown that the theory presented in [3] can be extended to include time dependence (on a suitable time scale, of course). In particular, therefore, we invoke an asymptotic method that is driven by the thin-shell approximation, which represents a minimal assumption for the atmosphere enveloping the Earth. (It is expedient also to take the shape of the Earth as ellipsoidal, and use almost-spherical coordinates; the role of this approximation is simply to adjust the nature of the background state, without affecting the leading-order unsteady description of the atmosphere: the uncoupling at this order is an important property of the underlying system. Thus this aspect of the problem is included in the general formulation, but ignored in the main thesis that we propound here.) As we found in our earlier work [3], the real surprise is that the thin-shell approximation, without recourse to any other assumptions about the nature of the flow, leads to a complete description of the dominant dynamic and thermodynamic elements that are needed to describe the atmosphere. This earlier work showed how standard steady-state models that are used for specific problems in the atmosphere (e.g. Ekman and geostrophic flows, Hadley cell circulation) can be recovered from one set of simple governing equations, and with many accurate interpretations that underlie the flow configurations (such as the precise nature of the heating that is needed to drive the motion). In the current study, our set of governing equations, (4.12)–(4.14), with (4.2) and (4.4), provide the basis for a systematic study of unsteady motion and, most particularly, of various modes of wave propagation in the troposphere.

The development presented here follows the path originated in [3]: a steady background state of the atmosphere (described by equations in which the velocity field appears at this same order) is perturbed by using the thin-shell parameter, with time included in the perturbation system. The time scale—a few hours—is that associated with the rotation of the Earth, which is the appropriate choice for reasonably large-scale wave-like motions of the atmosphere. The upshot is that we have a perturbation state that is unsteady, contains the main contributors to the dynamics and thermodynamics, as well as viscosity, compressibility, the Earth’s rotation and the underlying spherical geometry. In addition, the formulation allows for the identification of the heat sources that are needed to initiate and maintain the motion, and this connection is altogether transparent. One main advantage of the careful and systematic construction of a set of equations with a robust pedigree is that we can hope to develop numerical solutions of this simpler system, driven by reliable data. Our systematic approach is based on theoretical first principles, augmented by phenomenological insights (regarding the nature of the heat forcing), and avoids *ad hoc* assumptions. This conceptually coherent modelling brings to light the significant dependency of the atmospheric flow on the heat forcing, avoiding adjustments of model parameterizations that often mask underlying deficiencies of models, which are not derived systematically from the governing equations, a procedure that may fail to capture the relevant physical processes even if, due to parameter calibration, they might exhibit agreement between simulation and data.

Our equations admit solutions that are harmonic in time, and so the majority of our examples are based on solutions that possess this property. A familiar model for simple, oscillatory flows involves the Brunt–Väisälä frequency (*N*), so we started by investigating how such flows appear in our system. On the one hand, we see that *N*^2^ is directly and simply related to our forcing function (*F*_1_) and so its form for various flows can be interpreted in terms of the associated heat sources. However, the standard analysis, which leads to the usual dispersion relation involving *N*^2^, cannot be obtained in any systematic way (as explained in §5a(i)): we conclude that any such ‘derivations’, and conclusions based on the results, remain highly suspect. Fortunately, there are many other avenues that we can explore which lead to relevant and reliable results, with applications to the motion of the atmosphere.

The main thrust of the examples that we have presented here is for inviscid flows; viscous effects have been included in the formulation, but are weak (and relevant only in thin boundary layers) and are therefore set aside in much of what we present. The examples are intended to show what is possible; clearly, many other choices can be made, suitable for other flow configurations. (It should be noted that, in all cases, the existence of a background state, which varies in *ζ* = *gz* − (1/2) cos ^2^*θ*, ensures that there is always a variation in the vertical direction, with an associated distortion in the meridional direction.) The system that describes the dynamic-thermodynamic balance, with heat sources, admits solutions that are harmonic in *t*, *φ* and *ζ*, but not in *θ*: the dependence on *θ* necessarily involves some modulation in the meridional direction. With these points in mind, we have obtained a standing-wave solution, which oscillates in time only, as well as solutions that describe waves propagating in the meridional direction, and waves in the azimuthal direction. The former provides a model for waves that propagate towards the Equator (over the Arabian Sea); the latter is a solution of the type observed to be moving in an equatorial direction over the Indian Ocean. These solutions, and all similar ones, possess a vertical structure, provided in part by the existence of the background state, and all satisfying the condition *W* = 0 at the bottom of the atmosphere. We expect that many other examples, relevant to other modes and directions of propagation, with varying complexities of modulation in the meridional direction, can be identified; this is an area that needs further investigation.

Another example, which shows in greater depth the advantages of a general, all-embracing system of equations, arises when we consider waves that are trapped in the neighbourhood of the Equator. In contrast to the familiar approach, we need make no assumptions about the behaviour in the meridional direction: *f*- or *β*-plane approximations are altogether unnecessary. Simply by seeking a solution that imposes no motion in the meridional direction, together with a suitable heat forcing, leads to a solution that is restricted to a neighbourhood of the Equator, the trapping being represented by a power-law behaviour (and not exponential as in the simple theories). This example, perhaps more so than the previous ones described above, shows one of the significant advantages of our formulation: we can identify and interpret the heat sources required to maintain the motions.

One final example, discussed in some detail, shows how a time-averaged version of the equations, for periodic solutions, can be used to provide some general observations without recourse to the construction of explicit solutions. In particular, this procedure was applied to a description of the AEJ, showing how the temperature profile in the troposphere necessarily produces the observed properties of this atmospheric flow. Indeed, this calculation can be extended to include the African Easterly Wave as a time-dependent perturbation of the AEJ, although the details have not been pursued here.

There are many different types of unsteady motion in the atmosphere (including various instabilities) see [40]; we have chosen, as simple examples, a few modes of wave propagation (of which there are many) in this initial investigation. These have been chosen to demonstrate how details of the motion can be extracted and interpreted and, where appropriate, how general properties can be identified without the need for explicit solutions. In addition to these examples, we have also shown how the general time-dependent problem can be formulated, avoiding any assumptions about the nature of the time-like behaviour. Indeed, if the heat forcing is given, then the complete dynamical structure of the atmosphere can be calculated (at leading order). Furthermore, as with all the examples discussed here, the specification of the heat forcing, *F*_1_, can be used to provide the thermodynamic elements associated with the motion and lead to the interpretation of the required heat sources. All this, we emphasize, is based on a single set of simplified equations that are robustly connected to the underlying, governing equations for a fluid. Finally, we have outlined how the corresponding viscous problem can be solved, although the details are less readily accessible; more information on the viscous problem can be found in [3].

What we have developed and described here should be, we submit, the basis for the study of unsteady atmospheric flows. On the one hand, we have a set of equations derived carefully (using precise asymptotic methods) incorporating minimal simplifying assumptions; this system provides a reliable starting point for investigation, analysis and interpretation. On the other hand, particularly with the ready availability of extensive and reliable data, these equations can be used to generate numerical solutions, which, in turn, can become the seed for numerical studies of the original, full set of equations. In either case, we have shown how familiar results, and new results, can be obtained directly and systematically from the underlying governing equations. Furthermore, any simpler model should be tested against this system: can such models be obtained by making additional assumptions consistent with these equations, working altogether systematically? We submit that the only reliable validation of model equations is their (asymptotic) derivation from a set of general governing equations, along the lines that we have presented here.

## Data accessibility

All data are provided in full in the paper.
